# Childhood adversities and rate of adulthood all-cause hospitalization in the general population: A retrospective cohort study

**DOI:** 10.1371/journal.pone.0287015

**Published:** 2023-06-12

**Authors:** Asmita Bhattarai, Gina Dimitropoulos, Andrew G. M. Bulloch, Suzanne C. Tough, Scott B. Patten

**Affiliations:** 1 Department of Community Health Sciences, Cumming School of Medicine, University of Calgary, Calgary, AB, Canada; 2 Mathison Centre for Research & Education, University of Calgary, Calgary, AB, Canada; 3 Faculty of Social Work, University of Calgary, Calgary, AB, Canada; 4 Department of Psychiatry, Cumming School of Medicine, University of Calgary, Calgary, AB, Canada; 5 Department of Pediatrics, Cumming School of Medicine, University of Calgary, Calgary, AB, Canada; Ehime University Graduate School of Medicine, JAPAN

## Abstract

**Objective:**

The study examined the association between specific childhood adversities and rate of all-cause hospitalization in adulthood in a large sample of the general population and assessed whether adult socioeconomic and health-related factors mediate those associations.

**Methods:**

We used linked data available from Statistics Canada i.e., the Canadian Community Health Survey (CCHS-2005) linked to Discharge Abstract Database (DAD 2005–2017) and Canadian Vital Statistics Database (CVSD 2005–2017). CCHS-2005 measured self-reported exposure to childhood adversities, namely prolonged hospitalization, parental divorce, parental unemployment, prolonged trauma, parental substance use, physical abuse, and being sent away from home for wrongdoing, from a sample of household residents aged 18 years and above (n = 11,340). The number and causes of hospitalization were derived from linkage with DAD. Negative binomial regression was used to characterize the association between childhood adversities and the rate of hospitalization and to identify potential mediators between them.

**Results:**

During the 12-year follow-up, 37,080 hospitalizations occurred among the respondents, and there were 2,030 deaths. Exposure to at least one childhood adversity and specific adversities (except parental divorce) were significantly associated with the hospitalization rate among people below 65 years. The associations (except for physical abuse) were attenuated when adjusted for one or more of the adulthood factors such as depression, restriction of activity, smoking, chronic conditions, poor perceived health, obesity, unmet health care needs, poor education, and unemployment, observations that are consistent with mediation effects. The associations were not significant among those aged 65 and above.

**Conclusion:**

Childhood adversities significantly increased the rate of hospitalization in young and middle adulthood, and the effect was potentially mediated by adulthood socioeconomic status and health and health care access related factors. Health care overutilization may be reduced through primary prevention of childhood adversities and intervention on those potentially mediating pathways such as improving adulthood socioeconomic circumstances and lifestyle modifications.

## Introduction

Childhood adversities are a broad range of stressful experiences that may have occurred in one’s childhood, including various forms of abuse, neglect, and exposure to dysfunctional family situations such as parental substance use and parental marital conflict [1, 2]. Childhood adversities are highly prevalent and are unequally distributed across the sociodemographic profile, such as age, sex, and race [3–5]. Extensive literature suggests that childhood adversities affect one’s physiological and psychological development, leading to adverse health outcomes. Childhood adversities have been reported to be strongly associated with psychosocial adversities such as poor social engagement, unstable relationships; health risk behaviors such as smoking and substance use; chronic conditions such as depression, distress, and cancer; socioeconomic deprivation such as poor income and education, and even premature mortality in adulthood [2, 6–8]. These associations of childhood adversities with adverse physical, mental, and social health suggest that through these potentially mediating mechanisms, there may be an increased need for, and utilization of, health care services in adulthood among people reporting childhood adversities.

Existing studies have reported that exposure to childhood adversities is associated with increased health service use [4, 9–11]. For instance, a Canadian study of the general population reported that cumulative exposure to childhood adversities (including both childhood abuse and household dysfunction variables), termed adverse childhood experiences (ACEs), was significantly associated with increased health professional use, emergency department (ED) use, and general practitioner (GP) use. Also, the study reported that the strength of association was stronger with physical abuse and sexual abuse than with parental marital conflict [4], suggesting that childhood adversities may have a differential effect on health service use based on the type of adversity [4, 10]. This draws into question the commonly employed strategy of scoring adversity questionnaires using counts of positive responses.

Studies have also reported that childhood adversities are associated with suboptimal uptake of preventive health care services, such as not having a primary care provider, the absence of regular check-ups, and screening for cancer [12–15]. For instance, a cross-sectional study conducted in 2011 among non-institutionalized adults living in the United States found that physical abuse and sexual abuse were highly associated with lower odds of self-reports of receiving regular preventive care, adjusting for access to care [10]. Also, it has been reported that people with exposure to childhood adversities are less likely to receive quality medical care [14]. Negative health-seeking behavior, coupled with the health risk behaviors, may further increase the burden of unplanned or recurrent acute health service use among people with a history of childhood adversities.

The pattern of reduced uptake of preventive services and increased use of emergency department and health professional services suggests that childhood adversities may also be associated with increased hospitalizations. Hospitalization is a little explored area of health care utilization in relation to childhood adversities. Out of the few, one study of adult household residents conducted in 2015 in England reported that levels of self-reported acute health service use increased with an increase in the number of ACEs one was exposed to (which included adversities such as physical abuse, sexual abuse, and parental psychopathology), independent of sociodemographic factors [9]. However, the study used a cumulative score for ACEs, and thus effects of individual adverse experiences were not reported. Additionally, it has also been reported that hospitalizations among children with a history of child abuse and neglect tend to be longer, have more co-morbid diagnoses, and have double the cost of treatment compared to those without abuse [16].

Hence, studying hospitalization as an outcome becomes important to inform policy and practice around childhood adversities and health service use. All-cause hospitalization could be a measure of burden on health services and a comprehensive and objective measure of the impact of childhood adversities [17–20]. Health service utilization is generally linked to improved morbidity management, reduced mortality, and improved quality of life [9, 10, 21]. However, if the burden increases, it may affect the resources available and the quality of care delivery in hospitals [16, 22].

There are some methodological limitations in the existing literature examining the effect of childhood adversities on adverse health and health care utilization, such as the use of retrospective reports of adversities, being conducted in clinical samples posing the risk of selection bias, assessing outcomes through self-reports posing the risk of measurement bias, focusing on chronic health conditions leaving out infectious and autoimmune diseases, and use of cross-sectional designs limiting temporal clarity [1, 4, 9, 10, 16, 23]. There is a need for studies examining the association between childhood adversities and hospitalization in a representative sample of the general population, incorporating objectively measured outcome data and examining different types of childhood adversities. The recent availability of large population-based sample survey data (Canadian Community Health Survey, CCHS) linked with the national administrative database of hospitalizations (Discharge Abstract Database, DAD) and mortality (Canadian Vital Statistics Database, CVSD) enables us to address some of the above-mentioned gaps and examine the association longitudinally. Also, there is a general tendency to focus on the index hospitalization as a measure of outcome [20, 24], compared to the study of multiple admissions or length of stay, which may be more informative [20], an issue that we were also able to address in our analysis.

The study thus investigates the association between specific childhood adversities and the number of hospitalizations among adults in the general population. CCHS measures a wide range of adult sociodemographic variables and collects information on health status and determinants of health, which may be risk factors for increased hospitalizations and could be the target of public health interventions [10, 25–28]. The study also aims to examine whether adult physical, mental, and social health-related variables may mediate the association between childhood adversities and the rate of hospitalization.

## Methods

### Data sources

This retrospective cohort study used secondary data available from the linkage of the Canadian Community Health Survey (CCHS) 2005 to the Discharge Abstract Database (DAD) 2005–2017 and the Canadian Vital Statistics Database (CVSD) 2005–2017. The data are not publicly available, but access can be obtained through an application process and security clearance procedure.

The CCHS is a cross-sectional survey conducted by Statistics Canada every year, which collects information about general health status and health determinants from Canadians aged 12 and over. It is administered Canada-wide, i.e., across all provinces and territories, excluding only around 3% of the population, consisting of those living on Indian Reserves and settlements, full-time members of the Canadian Armed Forces, and residents of institutions, as well as some remote areas. The survey uses a complex sampling design: a stratified multistage sampling technique with several sampling frames leading to unequal selection probabilities and design effects due to clustering. To account for this, Statistics Canada provides a master weight and sets of replicate bootstrap weights used to produce valid estimates and correct standard errors from a representative sample of the Canadian population. The weights also include post-stratification adjustments and adjustments for non-response. The survey has a reasonable response rate, 84.9% at the household level and 92.9% at the individual level. Most of the data were collected in person using computer-assisted interviews. Further details of the survey can be found on the Statistics Canada website [29].

DAD is a nationally standardized database that records hospital admissions. These are extracts from the discharge summary and contain information on deaths, transfers, discharges, and the clinical diagnoses coded according to International Classification of Diseases-Canada (ICD-10-CA) system (coded by trained professionals, a valid and reliable measure) [24, 30]. The CVSD records all deaths and the ICD-9 and 10 codes for causes of death in Canada [31]. The details of the data linkage procedures can be found elsewhere [32]. Under Canadian tri-council guidelines (TCPS-2), data governed under specific legislation (as was the case here) do not require additional ethical review. Statistics Canada obtained written consent for participation and data sharing at data collection.

The sample comprised of adults aged 18 years and above in 2005 who consented to sharing and linkage of their data with other administrative databases, and which excluded proxy respondents. A proxy respondent is another household member who completed the interview if the intended participant was unable to respond due to poor physical or mental health status. The childhood adversity module was selected and administered by two provinces, Saskatchewan, and Manitoba. Around 2.3% of the eligible sample did not respond to the adversity questions. Hence, the total sample size for analysis was around 11,340 respondents at baseline, which was probabilistically linked to 37,080 hospitalizations and 2,030 deaths among the respondents prior to the record linkage date on December 31, 2017. Based on Statistics Canada data release guidelines, all the frequencies were rounded to the nearest 5. The derivation of the final sample from the overall CCHS 2005 sample is illustrated in S1 Fig.

### Measures

#### Exposure

The exposures of interest were the specific childhood adversities, namely prolonged hospitalization, parental divorce, prolonged parental unemployment, being “sent away from home for doing something wrong," problematic parental substance use, prolonged trauma, and physical abuse, as measured by CCHS 2005 through responses to a series of binary response questions in the “Childhood and Adult Stressors (CST)” module [29]. The questions referred to the time when they were children or teenagers before they moved out of the house.

A composite dichotomous variable called "at least one childhood adversity" was also created, representing exposure to one or more childhood adversities under study. The childhood adversity items were selected and adopted by Statistics Canada from the pool of stressful life events used in the work of Blair Wheaton et al. [33]. Statistics Canada has done field testing of the items, but they have not been formally validated.

#### Outcome

The primary outcome of interest was the number of all-cause hospitalizations during the follow-up period (January 2005 to December 2017). The CCHS interviews were conducted between January 2005 and December 2005. Hospitalizations before the interview date of the respondents were not included in the count to better clarify the temporality of the outcome in relation to the exposure. Two types of linked databases, event-based and person-based, were created for the descriptive and analytical outcomes.

The linkage of CCHS 2005 to DAD 2005–2017 resulted in an event-based dataset for the cohort under study. This means that hospitalized respondents had each row of observation representing an event of hospitalization, and for those without hospitalization, the hospitalization variables were missing (later coded as zero). The various main causes of hospitalization (both single and multiple admissions) were also explored, which were categorized as: causes related to the circulatory system (I00-I99), neoplasm (C00-D49), respiratory system (J00-J99), nervous system and sense organs (G00-G99, H00-H95), mental and behavioral (F01-F99), digestive system (K00-K95), endocrine system (E00-E89), infectious diseases (A00-B99), genitourinary system (N00-N99), reproductive system (O00-O9A), musculoskeletal system (M00-M99), injury and poisoning (S00-T88), factors affecting health status (Z00-Z99), not elsewhere defined (R00-R99) and others (blood, skin, congenital, perinatal, external causes), using the ICD-10 codes available in the DAD database [34].

For analysis of the number of hospitalizations, a person-based database was created, where each respondent had only one row of observations and included the number of hospitalizations calculated from the event-based data. A dichotomous variable for at least one hospitalization (yes/no) was also created based on the linkage. Exposure time was calculated as the duration between the CCHS interview date and the end of the follow-up period for those who remained alive until December 31, 2017 (the linkage date), and between the CCHS interview date and date of death among those who died during the follow-up period. Including exposure time in statistical models of count outcome allows us to consider the varying number of person-years contributed by the participants due to the differing duration of follow-up and loss to follow-up due to death and enables us to model rates per person-year. The log of exposure time is called the "offset term," which has a constant slope, i.e., the coefficient is restricted to 1 [35].

#### Covariates

*Potential modifiers and confounders*. We examined sociodemographic variables, namely, age, sex (male vs. female), race (white vs. non-white), and immigrant status (immigrant vs. non-immigrant) as potential modifiers and confounders [3–5, 10, 24]. Age was modeled as a continuous variable and a categorical variable (below 65 years vs. 65 years and above) as appropriate. People aged 65 and above represent a large proportion of hospitalized patients, tend to have more co-morbidities and their care pathways may be more intensive than younger population and hence the age cut-off of 65 was used [36].

*Potential mediators*. Based on the existing literature [10, 25–28], we examined various socioeconomic, lifestyle, and psychosocial factors as potential mediators. The variables are dichotomized for uniformity and simplified presentation of results and to comply with the minimum cell size criteria of Statistics Canada for vetting of results.

The characteristics were recategorized and analyzed as past 12 months smoking status (current smoker vs. not a current smoker), alcohol use (used alcohol in past 12 months vs. no alcohol use), restriction of activity (yes vs. no), physical inactivity (yes vs. no), perceived general health (fair-to-poor vs. good-to-excellent), perceived mental health (fair-to-poor vs. good-to-excellent), obesity according to body mass index (BMI) cut off of 30 (obese vs. non-obese), predicted probability of depression measured using Composite International Diagnostic Interview- Short Form (depressed vs. non-depressed using cut off representing 0.9 positive predictive value), living arrangement (living alone vs. living with partner/others), and unmet healthcare needs (yes vs. no). The presence of any chronic condition was included as a dichotomous variable, which was defined as the presence of at least one of the following conditions diagnosed by a health professional: arthritis, asthma, back problems, chronic lung disease, cataracts or glaucoma, cancer, Crohn’s disease, diabetes, epilepsy, heart disease, high blood pressure, migraine, stroke, thyroid disease, and peptic ulcer disease.

The variables representing adult socioeconomic status were marital status (never married vs. ever married), educational status (high school graduate vs. non-graduate), employment status (employed vs. not currently employed at the time of the baseline interview), and total household income (low or no income vs. high income based on cut-off $20,000), during the survey year 2005. The questionnaire and the definitions for derived variables are available on the Statistics Canada website [29].

### Statistical analysis

Data management and analysis used STATA software version 16 [37] at the Prairie Regional Data Centre (RDC) at the University of Calgary. In keeping with Statistics Canada guidelines, the estimates were weighted and bootstrapped as appropriate.

The descriptive statistics were reported as weighted percentages and 95% Confidence Intervals (CIs) for categorical variables, mean and 95% CI for continuous variables, and median and interquartile range (IQR) for count variables. The association between specific childhood adversities and covariates was examined using logistic regression, and the weighted and bootstrapped (500 iterations) estimates are reported as Odds Ratios (ORs) and 95% CIs.

To examine the differences in the expected number of all-cause hospitalizations per person-year across the exposure groups and covariates, Poisson regression analysis was performed first, which also included the log of exposure time as an offset [35]. Since we aim to examine the separate effects of childhood adversities, separate models were run for each specific childhood adversity, along with a composite variable representing one or more adversity. There was evidence of overdispersion in the Poisson models, as the observed variance was much higher than the mean across all childhood adversity variables (result not shown) [35]. Furthermore, according to a spike plot, there was no evidence of zero inflation (results not shown) [38]. Based on these observations, a negative binomial regression model [39], was used instead of Poisson regression to examine the association of childhood adversities and the covariates with the over dispersed number of hospitalizations. The models used the sampling weights provided by Statistics Canada and included the offset as a covariate, and the estimates were reported as Incident Rate Ratios (IRRs) and 95% CIs. IRRs represent the ratio of the incidence rate in the exposed group to the incidence rate in the unexposed group during the study period [40]. The negative binomial models could not use the bootstrapping procedure that Statistics Canada suggested, so the 95% CIs may not have fully addressed clustering in the sampling design [29]; specifically, the confidence intervals may be too narrow.

In all the associations, assessment of effect modification by age, sex, race, and immigrant status was done using interaction terms between those covariates and specific childhood adversities. A p-value of <0.05 for the interaction term was interpreted as evidence of effect modification. Confounding was assessed through the relative difference between unadjusted and adjusted estimates of associations for each covariate. Sex, race, and immigration status neither modified nor confounded the association between childhood adversities and the number of hospitalizations. However, age confounded most of the associations. Also, the interaction terms between age and some childhood adversities (prolonged trauma, physical abuse, and at least one adversity) were statistically significant. Hence, we decided to stratify the association between each childhood adversity and the number of hospitalizations by age group (cut off 65 years) and adjust for age in years in the stratified models to address the residual confounding present due to the broad age range within the groups.

Further, although a full mediation analysis could not be done with the available data, we examined whether adult physical, mental, and social health-related variables (associated with both rate of hospitalizations and specific childhood adversities) may have mediated the significant associations in the above analysis. For this, each potential mediating variable was adjusted for in the models of specific childhood adversities and age-adjusted number of hospitalizations. If the associations attenuated with these adjustments, the variable was interpreted as a potential mediator of the association between childhood adversities and the rate of hospitalizations.

## Results

The sample characteristics at baseline are presented in Table 1. Most of the sample was aged <65 years (84.05%), female (51.02%), white in race (84.08%), and non-immigrant (78.09%). Most of the sample had used alcohol in the past year (81.33%) and had at least one chronic condition (70.77%). Also, 24.05% of the sample were currently smoking, 12.17% had poor perceived general health, and 4.85% had poor perceived mental health. Moreover, 11.70% reported that they did not receive health care when needed in the past year. The median number of hospitalizations in the sample was 1 (IQR 0–3), and that among those hospitalized was 3 (IQR 1–5).

**Table 1:** Sample characteristics in the overall sample at baseline (CCHS 2005) (n = 11,340).

Characteristics	Categories	Proportion % (95% CI)
Age		46.14 (46.13,46.15)
Age group	<65 years	84.05 (84.03,84.06)
	> = 65 years	15.95 (15.94,15.97)
Sex	Male	48.98 (48.96,49.00)
	Female	51.02 (51.00,51.04)
Race	Non-white	15.92 (15.90,15.93)
	White	84.08 (84.07,84.10)
Immigrant status	Immigrant	21.91 (21.89,21.92)
	Non-immigrant	78.09 (78.08,78.11)
Marital status	Never married	22.62 (22.61,22.64)
	Ever married	77.38 (77.36,77.39)
Education status	HS graduate	76.63 (76.61,76.64)
	Not HS graduate	23.37 (23.36,23.39)
Employment status*	Currently employed	70.43 (70.41,70.45)
	Currently not employed	29.57 (29.55,29.59)
Household income	Low income	9.78 (9.77,9.79)
	Middle/high income	90.22 (90.21, 90.23)
Smoking	current smoker	24.05 (23.98,24.12)
	not current smoker	75.95 (75.88,76.02)
Chronic condition	has any chronic condition	70.77 (70.70,70.85)
	no chronic condition	29.23 (29.15,29.30)
Self-perceived general health	fair/poor	12.17 (12.12,12.22)
	good/excellent	87.83 (87.78,87.88)
Probability of depression	depressed	4.87 (4.82,4.92)
	not depressed	95.28 (95.23,95.33)
Obesity	obese	19.75 (19.69,19.82)
	non-obese	80.25 (80.18,80.31)
Physical inactivity	physically inactive	52.93 (52.85,53.01)
	not physically inactive	47.13 (47.05,47.21)
Alcohol use in past 12 months	used alcohol	81.33 (81.27,81.39)
	no alcohol use	18.67 (18.61,18.73)
Living arrangement	living alone	15.25 (15.19,15.30)
	living with partner/others	84.69 (84.63,84.74)
Restriction of activity	restricted	33.84 (33.77,33.92)
	not restricted	66.16 (66.08,66.23)
Perceived mental health	Fair/poor	4.85 (4.82,4.89)
	Good/excellent	95.15 (95.11,95.18)
Unmet healthcare needs	Yes	11.70 (11.65,11.75)
	No	88.30 (88.25,88.35)
At least one hospitalization	Yes	65.94 (65.86,66.01)
	No	34.00 (33.92,34.07)

*Employment status was measured among people aged 18–74 years

The characteristics of the overall CCHS sample aged 18 and above were like the characteristics of the study sample (Refer to S1 Table).

Table 2 presents the prevalence of at least one childhood adversity and specific childhood adversities in the sample. Around 46% of the sample reported exposure to at least one childhood adversity. The most common childhood adversity was prolonged trauma (18.35%), and the least reported was being sent away from home (2.40%).

**Table 2:** Prevalence of childhood adversities in the overall sample (CCHS 2005).

Childhood adversities	Prevalence % (95% CI)
Prolonged hospitalization	15.65 (15.59,15.71)
Parental divorce	11.97 (11.92,12.02)
Prolonged parental unemployment	9.34 (9.29,9.38)
Prolonged trauma	18.35 (18.28,18.41)
Problematic parental substance use	14.41 (14.35,14.47)
Physical abuse	7.40 (7.36,7.44)
Being sent away from home	2.40 (2.37,2.42)
At least one childhood adversity	45.75 (45.67,45.83)

Table 3 presents the proportion of various causes of hospitalization in the sample, calculated using the event-based data. For each diagnosis category, the numerator included the number of single or multiple hospitalizations with the specific diagnosis over the follow-up period, whereas the denominator included all the observations in the event-based data (i.e., the sum of no admissions and single and multiple admissions). Hence, the sum of the proportion of various main causes of hospitalization in Table 3 (88.7%) is not the same as the proportion of at least one hospitalization in person-based data, which was around 66%. The most common primary diagnoses for hospital admissions (including both single and multiple admissions) were causes related to the digestive system (14.88%).

**Table 3:** Distribution of most responsible diagnoses of all hospitalizations in the overall sample.

Main diagnoses (Including multiple admissions)	Proportion % (95% CI)
Circulatory causes	8.27 (8.24,8.29)
Neoplasm	8.33 (8.31,8.36)
Respiratory causes	4.24 (4.22,4.26)
Nervous and sense organs cause	7.78 (7.76,7.81)
Mental causes	1.43 (1.42,1.44)
Digestive causes	14.88 (14.85,14.92)
Endocrine causes	1.25 (1.24,1.26)
Infectious causes	1.08 (1.07,1.09)
Genitourinary causes	7.10 (7.08,7.12)
Reproductive causes	5.47 (5.45,5.49)
Musculoskeletal causes	6.13 (6.11,6.16)
Not elsewhere defined	5.34 (5.32,5.36)
Injury	4.53 (4.51,4.55)
Factors affecting health status	11.26 (11.23,11.28)
Others (blood, skin, congenital, perinatal, external)	1.65 (1.64,1.66)
No admission	11.26 (11.23,11.28)

Age in years (crude IRR 1.03; 95% CI 1.03,1.03), female sex (age-adjusted IRR 1.22; 95% CI 1.13,1.32), non-white race (age-adjusted IRR 0.71; 95% CI 0.62,0.81), and immigrant status (age-adjusted IRR 0.84; 95% CI 0.73,0.97) were associated with rate of hospitalization. The unadjusted and above-mentioned covariate-adjusted associations between childhood adversities and the rates of hospitalizations are presented in Table 4. Exposure to at least one childhood adversity and most of the specific childhood adversities (except parental divorce) were significantly associated with an increased rate of hospitalization when adjusted for age, sex, race, and immigrant status. The adjusted estimates were generally larger than the unadjusted estimates of IRR, and further analysis suggested that the confounding was due to age, see Table 4. Hence, in all subsequent analyses, the estimates were adjusted for age (in years).

**Table 4:** Association between childhood adversities and rate of hospitalizations.

Childhood adversities	Crude IRR (95% CI)	Age-adjusted IRR (95% CI)	Covariates adjusted IRR (95% CI) #
Prolonged hospitalization	1.32 (1.21,1.44)	1.24 (1.14,1.36)	1.27 (1.16,1.39)
Parental divorce	0.74 (0.65,0.84)	1.08 (0.95,1.23)	1.08 (0.95,1.24)
Prolonged parental unemployment	1.03 (0.90,1.17)	1.27 (1.12,1.44)	1.23 (1.08,1.41)
Prolonged trauma	1.07 (0.97,1.18)	1.23 (1.12,1.36)	1.19 (1.08,1.32)
Problematic parental substance use	1.05 (0.94,1.17)	1.26 (1.13,1.41)	1.23 (1.09,1.39)
Physical abuse	1.11 (0.99,1.24)	1.44 (1.27,1.63)	1.37 (1.21,1.55)
Sent away from home	0.82 (0.67,1.00)	1.28 (1.05,1.56)	1.30 (1.07,1.58)
At least one childhood adversity	1.08 (1.00,1.16)	1.24 (1.15,1.34)	1.22 (1.13,1.32)

# IRR adjusted for age, sex, race, and immigration status

The interaction term between age and at least one adversity, physical abuse, and prolonged trauma was statistically significant (interaction terms p-value <0.05). Therefore, we stratified the estimates for all the childhood adversities and rates of hospitalization according to the age group (<65 years vs. > = 65 years) (Table 5) for consistency. Sex, race, and immigrant status did not modify (interaction terms p-value >0.05) nor confound the association between childhood adversities and hospitalization rate in the stratified analyses as well. There was a significantly higher age-adjusted hospitalization rate among people who reported all exposures other than parental divorce among those below 65 years. None of the associations were significant among those aged 65 years and above.

**Table 5:** Association between childhood adversities and rate of hospitalizations stratified by age group.

Childhood adversities	Among those <65 years old		Among those > = 65 years old	
	Age-adjusted IRR (95% CI)	Age, sex, race, immigration adjusted IRR (95% CI)	Age adjusted IRR (95% CI)	Age, sex, race, immigration adjusted IRR (95% CI)
Prolonged hospitalization	1.30 (1.17,1.44)	1.34 (1.20,1.49)	1.11 (0.98,1.26)	1.11 (0.98,1.25)
Parents divorced	1.08 (0.93,1.24)	1.09 (0.94,1.27)	0.97 (0.75,1.26)	1.02 (0.78,1.33)
Prolonged parental unemployed	1.29 (1.12,1.49)	1.25 (1.08,1.45)	1.05 (0.86,1.28)	1.05 (0.86,1.28)
Prolonged trauma	1.31 (1.17,1.46)	1.25 (1.10,1.41)	0.92 (0.81,1.04)	0.98 (0.86,1.11)
Problematic parental substance use	1.32 (1.17,1.49)	1.30 (1.13,1.48)	1.10 (0.90,1.34)	1.09 (0.89,1.33)
Physical abuse	1.51 (1.33,1.72)	1.42 (1.24,1.62)	0.93 (0.74,1.18)	0.99 (0.78,1.24)
Sent away from home	1.25 (1.02,1.53)	1.28 (1.05,1.57)	-*	-*
At least one childhood adversity	1.31 (1.20,1.44)	1.29 (1.18,1.42)	1.01 (0.91,1.12)	1.03 (0.93,1.14)

*Estimates for being sent away not presented due to Statistics Canada data release restrictions (low cell count in the old age group with exposure and outcome)

Most of the adulthood physical, mental, and social health-related variables were associated with the rate of hospitalization among people aged less than 65 years, except for physical inactivity, living arrangement, and never married status (Table 6).

**Table 6:** Association between potential confounders and mediators and number of hospitalizations among those aged <65.

Sample characteristics	Age-adjusted IRR (95% CI)
Current smoking status	1.20 (1.09,1.32)
Alcohol use	0.88 (0.78,0.99)
Chronic condition	1.72 (1.56,1.89)
Poor perceived general health	1.84 (1.61,2.09)
Physical inactivity	1.10 (1.00,1.20)
Restriction of activity	1.61 (1.46,1.77)
Depression	1.44 (1.16,1.77)
Obesity	1.31 (1.17,1.46)
Living arrangement	1.09 (0.99,1.19)
Never married status	1.02 (0.91,1.14)
Low education	1.31 (1.17,1.46)
Low income	1.54 (1.35,1.75)
Currently not employed	1.44 (1.30,1.61)
Poor perceived mental health	1.57 (1.30,1.90)
Unmet healthcare needs	1.28 (1.13,1.45)

These factors associated with hospitalizations, except alcohol use, were also associated with at least one childhood adversity and one or more specific types of childhood adversities (Refer to S2 Table). Based on the association with both exposure and outcome, the variables namely, smoking, chronic condition, poor perceived general health, poor perceived mental health, restriction of activity, depression, obesity, low education, low income, current unemployment, and unmet health care needs were examined as potential mediators. The age-adjusted IRRs and 95% CI for the association between all the childhood adversities and rate of hospitalization, further adjusted for the variables mentioned above one at a time, among those aged <65 years, are reported in the S3 Table.

Table 7 presents only the associations which were attenuated with adjustment for covariates. The association of prolonged hospitalization, prolonged parental unemployment, and parental problematic substance use in childhood with the rate of hospitalization was attenuated when adjusted for depression in adulthood. Restriction of activity weakened the association between prolonged trauma and the rate of hospitalization. Furthermore, current smoking status, presence of chronic conditions, poor perceived general health, restriction of activity, obesity, poor perceived mental health, low education, unemployment, and unmet health care needs attenuated the association between being sent away from home and the rate of hospitalization. The association of physical abuse did not attenuate with any of the adjustments.

**Table 7:** Association between adversities and age-adjusted number of hospitalizations adjusted for each of the potential modifiers among those aged 18 to 64 years.

	Prolonged hospitalization	Parents unemployed	Prolonged trauma	Parents substance use	Sent away
	Age-adjusted IRR (95% CI)				
	1.30 (1.17,1.44)	1.29 (1.12,1.49)	1.31 (1.17,1.46)	1.32 (1.17,1.49)	1.25 (1.02,1.53)
Potential mediators adjusted for	Age and mediator adjusted IRR (95% CI) for the rate of hospitalizations				
Current smoking status	-	-	-	-	1.18 (0.96,1.45)
Chronic condition	-	-	-	-	1.20 (0.98,1.47)
Poor Perceived health	-	-	-	-	1.19 (0.96,1.47)
Poor perceived mental health	-	-	-	-	1.20 (0.98,1.47)
Depression	1.15 (1.00,1.32)	1.17 (0.94,1.45)	-	1.16 (0.97,1.38)	-
Restriction of activity	-	-	1.19 (1.07,1.33)	-	1.15 (0.92,1.43)
Obesity	-	-	-	-	1.21 (0.98,1.50)
Unmet health care needs	-	-	-	-	1.21 (0.99,1.49)
Low education	-	-	-	-	1.17 (0.95,1.45)
Current unemployment	-	-	-	-	1.22 (0.99,1.49)

## Discussion

In this study of a large population-based sample, we found that childhood adversities were associated with hospitalizations among young adults and middle-aged adults (aged less than 65 years). Exposure to at least one childhood adversity had a significant association with the rate of hospitalization in that age group. Also, exposure to most of the specific childhood adversity items were significantly associated with an age-adjusted rate of all-cause hospitalization among people aged less than 65 years, and they had a similar moderate strength of association. The age-adjusted associations were independent of other sociodemographic variables, namely, sex, race, and immigrant status. Adulthood health-related factors such as depression, distress, restriction of activity, smoking, presence of a chronic condition, obesity, perceived physical and mental health, and perceived unmet health care needs, as well as low education and current unemployment, attenuated the association of some of the childhood adversities with all-cause hospitalizations when they were adjusted. The associations were not significant among those aged 65 and older. Around 46% of the sample reported exposure to at least one childhood adversity, consistent with previous studies that reported childhood adversities are highly prevalent [41, 42].

The association between childhood adversities and the rate of hospitalization has not been studied extensively. Most of the studies examining the effect of childhood adversities on health service utilization have reported associations with increased service uptake, such as emergency department visits, general practitioners’ visits, the number of doctor’s office visits, and preference of hospitals as a source of medical care [9, 23] and reduced uptake of preventive health services [4, 10], thus limiting our ability to compare our study findings. The few studies which have reported hospitalizations as the outcome have reported significant associations with childhood adversities, supporting the findings of our study. A study of the general population from England reported that people who had exposure to 4 or more childhood adversities (including abuse and household dysfunction) had significantly higher odds of any overnight hospital stay than those with no exposure (OR 2.67) [9]. Another population-based cohort study (children aged 0–15 years) with a follow-up of 24 years in Denmark reported that hospitalization rates during childhood and young adulthood were significantly higher among the high childhood adversity groups (dimensions such as deprivation, negative family functioning, and loss or threat within the family) compared to those with low childhood adversity [43]. The study included childhood adversities such as prolonged parental unemployment and parental substance abuse in their adversity groups, which were included in our study as well. However, these studies did not report the specific effect of individual childhood adversities, and the sample was relatively young. Our study examined and reported the effect of specific childhood adversities on the rate of hospitalizations in a wide age range in adulthood. The strength of associations of exposure to any childhood adversity and most of the specific childhood adversities with the rate of hospitalizations were not very heterogeneous. However, the non-significant association of parental divorce supports the methodological approach of our study of examining separate effects of childhood adversities in relation to adverse health outcomes.

The study found a significant association of specific childhood adversities with the rate of hospitalization among young and middle-aged adults but not among the elderly. This non-significant finding related to health care use among the elderly is not commonly reported because most studies have reported the associations in an overall young or middle-aged sample on average [10, 43]. A study of household adult residents aged 18 to 69 years in England reported that overnight hospital stay remained associated with childhood adversities score among the elderly (60–69 years), which contrasts with our findings [9]. However, it should be noted that the elderly group in the study mentioned above included a comparatively younger group in the elderly population compared to our study (aged 65 to 102 years), which may explain the difference in results for hospitalization, and a reduction of effect in elderly population may have been seen had a higher age range been included in the English study. The non-significant association for the number of ED visits and GP visits in their elderly group supports our finding of effect modification by age.

The non-significant association in the elderly may have been contributed by low reporting of childhood adversities by the elderly compared to younger people, which we found in our study (Refer to S4 Table). Studies have reported that younger people are more likely to report childhood trauma because they may be more informed and comfortable acknowledging the events to be traumatic given the ongoing dialog about these issues in recent years, whereas older people may still be reluctant to report or may have modified/minimized the recollection of such adversities [44–47]. Another explanation for low reporting by the elderly could be that people with childhood adversities may have died prematurely [44, 48], and some of those living may not recall those adversities as memories tend to fade with time [49]. Also, age-related illnesses and comorbidities increase with old age among people without childhood adversities as well [2]. Hence, based on these mechanisms underplay, the significant association among young and middle-aged adults and non-significant association among the elderly may suggest that the association between childhood adversities and hospitalization attenuate over time.

Another important finding in the study was the potential mediation by adult socioeconomic status, lifestyle, and health-related factors in the association between childhood adversities and the rate of hospitalizations among young and middle-aged adults. In the study, consistent with the findings of previous studies [24, 50, 51], these factors were significantly associated with most of the specific childhood adversities and rate of hospitalization, supporting the mediation hypothesis. Although the potential mediators were measured at baseline along with childhood adversity items and the causality cannot be entirely ascertained with this study design, since most of those variables refer to the past year, we can be slightly more confident that childhood adversities preceded the mediating variables. Depression, poor perceived physical and mental health, presence of chronic conditions, smoking, restriction of activity, low education, and perceived unmet health care needs attenuated the association of some specific childhood adversities, such as prolonged trauma, parental substance use, parental unemployment, being sent away from home, and prolonged hospitalization further suggesting that these factors may fall on the causal pathway leading to hospitalization. For instance, the depression variable, referring to a higher probability of depression, exhibited a pattern consistent with mediation across a range of childhood adversity types (prolonged hospitalization, parental unemployment, and parental substance use). The lack of association between these adversities and rate of adult hospitalization after adjustment for depression suggests that depression may fully mediate the association. Thus, preventing or minimizing depression among people with lived experience of such childhood adversities may substantially reduce hospitalizations [6, 52, 53].

In our study, the association of the item “being sent away from home for doing something wrong” was mediated by a greater number of the mediators (poor perceived physical and mental health, presence of chronic conditions, smoking, restriction of activity, obesity, low education, current unemployment, and perceived unmet health care) than other childhood adversities. People reporting this specific adversity also had a disproportionately higher burden of mental health and injury-related morbidity, suggesting that this specific item may have a more extensive effect on adult health and health service utilization. Being sent away from home is not an extensively studied childhood adversity, and thus future studies should provide more attention to this specific adverse childhood event.

The potential mediating effect of perceived unmet health care needs is also an important finding. The reasons for the perception of health care needed but not received were broad in the survey and included barriers from both the demand and supply side of health care such as service not available in the area and at the time required, long waiting times, and inability to access care due to health problem. In any case, the findings suggest that people with childhood adversities may have barriers to accessing primary health care [10, 54, 55], which may lead to unplanned or recurrent, otherwise preventable hospitalizations. The public health implication of these findings would be that these adult health and lifestyle-related factors may be the target of public health interventions to prevent health service overutilization and reduce the burden on the health system. Preventive care is known to significantly reduce hospitalizations [17, 18].

The study has several strengths and limitations to consider. The study used the data from a large national health survey of the general population. The linkage of CCHS data to DAD and CVSD databases provided the opportunity to follow up the sample for more than ten years. The linkage also enabled assessment of the rate of hospitalization through consideration of the differential contribution to the risk pool (due to death and differential recruitment date). The rate provides information on the rapidity of the occurrence of health events at various times [40]. Excluding adulthood hospitalizations before the survey date in the analysis helped better explain the directionality of the association under study. The objective measurement of hospitalization data instead of self-reported hospitalization is a strength, preventing recall bias in outcome measurement. Assessment of all-cause hospitalization enabled us to include a range of diagnosed mental and physical health conditions, both acute and chronic and both infectious and non-infectious, as opposed to the approach taken by many previous studies of focusing only on specific medical conditions or chronic conditions. The childhood adversity items were selected and adopted by Statistics Canada from the pool of life stressors put forward by Blair Wheaton [33], and the validity of the items has not been assessed or published. However, Statistics Canada has conducted field testing of the items while adopting them in the survey, thus providing credibility to the childhood adversity items. Also, this wide range of childhood adversities, a public health issue, need to be studied in the general population.

Childhood adversities were measured retrospectively through self-reports. Given the sensitive nature of the issues and the stigma attached, childhood adversity items may be under-reported, introducing misclassification bias. The bias may be differential or non-differential, based on whether the recall or reporting was influenced by adult health status [56]. However, the comparison of association of prospectively and retrospectively measured childhood adversities with many adulthood adverse health outcomes have been reported to be similar [56, 57]. The study included a brief list of childhood adversities mostly related to household dysfunction and one child abuse item, i.e., physical abuse. Future studies should include a more comprehensive list of other important forms of childhood adversities such as sexual abuse, emotional abuse, neglect, and bullying [4, 9]. Additionally, there is a possibility of uncontrolled confounding in the study by the unmeasured confounders such as household socioeconomic status in childhood, family adversity such as harsh discipline, low parental education, and parental psychopathology [21, 58]; which future studies should incorporate in their analyses of childhood adversities and health care utilization, to rule out alternate causality. The sample characteristics of the study were similar to the characteristics of the overall CCHS sample aged 18 and above at the national level. However, the generalization of study findings across all provinces should be made with caution, as the study used data from two provinces, and hospitalization policies may vary across provinces due to separately administered health systems.

## Conclusion

The study contributes to the childhood adversity literature by quantifying the association between specific childhood adversities and the burden of hospitalization. Most of the childhood adversities were significantly associated with the rate of hospitalization in young and middle adulthood, and the effect was potentially mediated by adulthood socioeconomic, health status, and health care access-related factors. The findings highlight the importance of all levels of prevention measures in addressing the effect of childhood adversities on hospital overutilization. In addition to primary prevention of childhood adversities (such as through family therapy), there is a need for mitigation of the associated psychosocial and disease burden among the survivors of childhood adversities, such as through public health interventions on the improvement of adulthood socioeconomic circumstances and lifestyle modifications [24, 41], to improve outcomes and reduce demand on health system caused by increased hospitalizations. Addressing barriers to accessing primary care may also be helpful to reduce the impact of childhood adversities [17, 18]. The non-significant association in the elderly population may suggest that the effect of life stressors such as childhood adversities on hospitalization may gradually become less apparent with increasing age.

## Supporting information

S1 Fig Derivation of study sample from CCHS 2005. (DOCX)

S1 Table Characteristics of the CCHS 2005 respondents in the full sample (18 years and older) and the study sample. (DOCX)

S2 Table Association between childhood adversities and potential mediators (which are associated with rate of hospitalization) (adversities as exposure and covariates as outcomes). (DOCX)

S3 Table Association between childhood adversities and age adjusted number of hospitalizations adjusted for each of the potential mediator among those aged 18 to 64 years. (DOCX)

S4 Table Prevalence of childhood adversities stratified by age groups. (DOCX)
