# Supplementary material for: Childhood adversities and rate of adulthood all-cause hospitalization in the general population: A retrospective cohort study
Source: PLoS One. 2023 Jun 12;18(6):e0287015. doi: 10.1371/journal.pone.0287015 (PMC10259787; doi:10.1371/journal.pone.0287015)
Supplement: S1 Fig — (DOCX) [file pone.0287015.s001.docx]

# **Childhood adversities and rate of all-cause hospitalization in adulthood in the general population: a retrospective cohort study**

**Fig 1: Derivation of study sample from CCHS 2005***

CCHS 2005

Overall sample aged 12 years and above

N=109,965

CCHS 2005 participants identified in the CVSD database

N= 2,030

Sample aged 18 and above who were non-proxy and responded to adversity questions

N= 11,340

Sample aged 18 and above and non-proxy who were administered childhood adversity questions

(Saskatchewan and Manitoba)

N=11,605

Number of hospitalizations linked in DAD 2005-2017

N= 37,080

* In keeping with Statistics Canada data release guidelines, all estimates are weighted and rounded to the nearest 5.

Sample aged 18 years and above

N=99,680
