# Supplementary material for: Childhood adversities and rate of adulthood all-cause hospitalization in the general population: A retrospective cohort study
Source: PLoS One. 2023 Jun 12;18(6):e0287015. doi: 10.1371/journal.pone.0287015 (PMC10259787; doi:10.1371/journal.pone.0287015)
Supplement: S1 Table — (DOCX) [file pone.0287015.s002.docx]

# **Childhood adversities and rate of all-cause hospitalization in adulthood in the general population: a retrospective cohort study**

**S1 Table: Characteristics of the CCHS 2005 respondents in the full sample (18 years and older) and the study sample**

| **Characteristics** | **Categories** | **Full sample (N=99,680)**  **% (95% CI)** | **Study sample (N=11,340)**  **% (95% CI)** |
| --- | --- | --- | --- |
| Age group | <65 years | 84.05 (84.03,84.06) | 82.60 (82.54,82.66) |
|  | >=65 years | 15.95 (15.94,15.97) | 17.33 (17.27,17.39) |
| Sex | Male | 48.98 (48.96,49.00) | 48.41 (48.33,48.49) |
|  | Female | 51.02 (51.00,51.04) | 51.59 (51.51,51.67) |
| Race | Non-white | 15.92 (15.90,15.93) | 12.97 (12.91,13.02) |
|  | White | 84.08 (84.07,84.10) | 86.97 (86.91,87.02) |
| Immigrant status | Immigrant | 21.91 (21.89,21.92) | 11.62 (11.56,11.67) |
|  | Non-immigrant | 78.09 (78.08,78.11) | 88.38 (88.33,88.44) |
| Marital status | Never married | 22.62 (22.61,22.64) | 21.28 (21.21,21.34) |
|  | Ever married | 77.38 (77.36,77.39) | 78.72 (78.66,78.79) |
| Education status | HS graduate | 76.63 (76.61,76.64) | 72.81 (72.74,72.88) |
|  | Not HS graduate | 23.37 (23.36,23.39) | 27.19 (27.12,27.26) |
| Current employment status | Employed | 70.43 (70.41,70.45) | 75.09 (75.02,75.16) |
|  | Unemployed | 29.57 (29.55,29.59) | 24.91 (24.84,24.98) |
| Household income | Low income | 9.78 (9.77,9.79) | 11.58 (11.52,11.63) |
|  | Middle/high income | 90.22 (90.21, 90.23) | 88.50 (88.44,88.55) |
