# Supplementary material for: Childhood adversities and rate of adulthood all-cause hospitalization in the general population: A retrospective cohort study
Source: PLoS One. 2023 Jun 12;18(6):e0287015. doi: 10.1371/journal.pone.0287015 (PMC10259787; doi:10.1371/journal.pone.0287015)
Supplement: S2 Table — (DOCX) [file pone.0287015.s003.docx]

# **Childhood adversities and rate of all-cause hospitalization in adulthood in the general population: a retrospective cohort study**

**S2 Table: Association between childhood adversities and potential mediators (which are associated with rate of hospitalization) (adversities as exposure and covariates as outcomes)**

| **Potential mediators** | **Prolonged hospitalization** | **Parents unemployed** | **Prolonged trauma** | **Parents substance use** | **Physical abuse** | **Sent away** | **At least one adversity** |
| --- | --- | --- | --- | --- | --- | --- | --- |
|  | **OR (95% CI)** | | | | | | |
| Smoking | 1.39 (1.17,1.65) | 1.61 (1.32,1.97) | 1.45 (1.23,1.70) | 1.84 (1.55,2.18) | 2.33 (1.86,2.91) | 5.28 (3.66,7.62) | 1.80 (1.59,2.05) |
| Alcohol use | 0.83 (0.68,1.00) | 0.97 (0.75,1.25) | 1.04 (0.86,1.24) | 1.18 (0.95,1.46) | 1.13 (0.88,1.45) | 1.46 (0.81,2.62) | 1.13 (0.98,1.32) |
| Chronic condition | 2.06 (1.69,2.50) | 1.02 (0.81,1.28) | 1.64 (1.37,1.95) | 1.32 (1.11,1.58) | 2.39 (1.78,3.21) | 1.11 (0.70,1.76) | 1.52 (1.34,1.74) |
| Perceived health | 1.85 (1.55,2.21) | 1.38 (1.05,1.80) | 1.48 (1.23,1.79) | 1.30 (1.04,1.62) | 1.94 (1.52,2.48) | 1.71 (1.08,2.72) | 1.55 (1.32,1.82) |
| Restriction of activity | 1.76 (1.51,2.05) | 1.21 (0.98,1.49) | 2.04 (1.75,2.39) | 1.21 (1.02,1.42) | 2.01 (1.62,2.49) | 1.89 (1.27,2.81) | 1.59 (1.40,1.80) |
| Depression | 1.69 (1.14,2.52) | 3.37 (2.26,5.04) | 4.00 (2.93,5.45) | 2.41 (1.65,3.53) | 4.80 (3.35,6.87) | 3.32 (1.62,6.80) | 2.94 (2.08,4.15) |
| Obesity | 1.42 (1.19,1.71) | 1.11 (0.88,1.39) | 0.93 (0.78,1.10) | 1.19 (0.99,1.43) | 1.26 (0.99,1.59) | 1.61 (1.03,2.50) | 1.24 (1.09,1.42) |
| Education | 1.48 (1.27,1.73) | 1.14 (0.94,1.39) | 1.15 (0.98,1.35) | 1.13 (0.96,1.34) | 1.52 (1.22,1.89) | 1.86 (1.28,2.72) | 1.17 (1.04,1.31) |
| Income | 1.15 (0.96,1.38) | 1.30 (1.02,1.65) | 1.45 (1.22,1.74) | 1.16 (0.94,1.44) | 1.84 (1.43,2.37) | 1.78 (1.14,2.79) | 1.19 (1.03,1.38) |
| Employment | 1.20 (1.01,1.43) | 1.21 (0.96,1.52) | 1.41 (1.20,1.66) | 0.90 (0.76,1.07) | 1.48 (1.17,1.87) | 1.09 (0.73,1.62) | 1.15 (1.01,1.31) |
| Perceived mental health | 1.64 (1.24,2.15) | 1.53 (1.05,2.22) | 2.53 (1.95,3.28) | 1.30 (0.98,1.74) | 3.53 (2.55,4.89) | 2.67 (1.40,5.09) | 2.04 (1.56,2.66) |
| Unmet health care needs | 1.34 (1.08,1.66) | 1.68 (1.27,2.22) | 2.50 (2.04,3.07) | 1.50 (1.20,1.87) | 2.25 (1.72,2.94) | 2.58 (1.61,4.15) | 1.98 (1.66,2.36) |
