# Supplementary material for: Childhood adversities and rate of adulthood all-cause hospitalization in the general population: A retrospective cohort study
Source: PLoS One. 2023 Jun 12;18(6):e0287015. doi: 10.1371/journal.pone.0287015 (PMC10259787; doi:10.1371/journal.pone.0287015)
Supplement: S3 Table — (DOCX) [file pone.0287015.s004.docx]

# **Childhood adversities and rate of all-cause hospitalization in adulthood in the general population: a retrospective cohort study**

**S3 Table: Association between childhood adversities and age adjusted number of hospitalizations adjusted for each of the potential mediator among those aged 18 to 64 years**

| **Potential mediators adjusted** | **Age and mediator adjusted IRR (95% CI) for number of hospitalizations** | | | | | | |
| --- | --- | --- | --- | --- | --- | --- | --- |
|  | **Prolonged hospitalization** | **Parents unemployed** | **Prolonged trauma** | **Parents substance use** | **Physical abuse** | **Sent away** | **At least one adversity** |
| Smoking | 1.28 (1.15,1.43) | 1.27 (1.11,1.47) | 1.29 (1.15,1.45) | 1.30 (1.15,1.47) | 1.48 (1.30,1.69) | 1.18 (0.96,1.45) | 1.29 (1.18,1.41) |
| Chronic condition | 1.22 (1.10,1.35) | 1.28 (1.11,1.48) | 1.22 (1.09,1.36) | 1.28 (1.13,1.44) | 1.39 (1.22,1.57) | 1.20 (0.98,1.47) | 1.24 (1.14,1.36) |
| Poor Perceived health | 1.25 (1.12,1.39) | 1.25 (1.08,1.44) | 1.25 (1.12,1.38) | 1.28 (1.14,1.44) | 1.44 (1.27,1.64) | 1.19 (0.96,1.47) | 1.26 (1.15,1.38) |
| Restriction of activity | 1.23 (1.11,1.37) | 1.26 (1.09,1.45) | 1.19 (1.07,1.33) | 1.28 (1.13,1.44) | 1.39 (1.22,1.58) | 1.15 (0.92,1.43) | 1.24 (1.14,1.36) |
| Depression | 1.15 (1.00,1.32) | 1.17 (0.94,1.45) | 1.33 (1.12,1.58) | 1.16 (0.97,1.38) | 1.43 (1.22,1.68) | 1.38 (1.01,1.90) | 1.20 (1.06,1.35) |
| Obesity | 1.30 (1.16,1.45) | 1.26 (1.09,1.46) | 1.31 (1.17,1.46) | 1.32 (1.17,1.49) | 1.51 (1.33,1.71) | 1.21 (0.98,1.50) | 1.31 (1.20,1.44) |
| Low education | 1.27 (1.14,1.41) | 1.25 (1.08,1.44) | 1.28 (1.15,1.43) | 1.29 (1.14,1.45) | 1.46 (1.29,1.65) | 1.17 (0.95,1.45) | 1.28 (1.17,1.40) |
| Low income | 1.28 (1.15,1.43) | 1.20 (1.03,1.41) | 1.24 (1.11,1.40) | 1.30 (1.14,1.47) | 1.41 (1.24,1.61) | 1.24 (1.01,1.53) | 1.29 (1.18,1.42) |
| Unemployment | 1.27 (1.15,1.40) | 1.24 (1.08,1.43) | 1.25 (1.12,1.40) | 1.31 (1.16,1.48) | 1.43 (1.27,1.62) | 1.22 (0.99,1.49) | 1.28 (1.17,1.40) |
| Poor perceived mental health | 1.28 (1.15,1.42) | 1.28 (1.11,1.47) | 1.28 (1.13,1.42) | 1.31 (1.16,1.48) | 1.45 (1.28,1.63) | 1.20 (0.98,1.47) | 1.29 (1.18,1.41) |
| Unmet health care needs | 1.29 (1.16,1.43) | 1.28 (1.10,1.48) | 1.28 (1.14,1.43) | 1.31 (1.16,1.49) | 1.48 (1.30,1.60) | 1.21 (0.99,1.49) | 1.30 (1.18,1.42) |
