# Supplementary material for: Childhood adversities and rate of adulthood all-cause hospitalization in the general population: A retrospective cohort study
Source: PLoS One. 2023 Jun 12;18(6):e0287015. doi: 10.1371/journal.pone.0287015 (PMC10259787; doi:10.1371/journal.pone.0287015)
Supplement: S4 Table — (DOCX) [file pone.0287015.s005.docx]

# **Childhood adversities and rate of all-cause hospitalization in adulthood in the general population: a retrospective cohort study**

**S4 Table: Prevalence of childhood adversities stratified by age groups**

| **Childhood adversities** | **Categories** | **% (95% CI)** | |
| --- | --- | --- | --- |
|  |  | **<65 years** | **>=65 years** |
| Prolonged hospitalization | yes | 15.27 (15.20,15.33) | 17.44 (17.30,17.59) |
|  | no | 84.65 (84.59,84.72) | 82.56 (82.41,82.70) |
| Parents divorced | yes | 13.52 (13.46,13.58) | 4.60 (4.52,4.68) |
|  | no | 86.48 (86.42,86.54) | 95.40 (95.32,95.48) |
| Prolonged parental unemployment | yes | 9.91 (9.86,9.96) | 6.59 (6.49,6.69) |
|  | no | 90.09 (90.04,90.14) | 93.41 (93.31,93.51) |
| Prolonged trauma | yes | 18.95 (18.88,19.02) | 15.44 (15.31,15.58) |
|  | no | 81.05 (80.98,81.12) | 84.56 (84.42,84.69) |
| Problematic parental substance use | yes | 14.41 (14.35,14.47) | 5.79 (5.70,5.88) |
|  | no | 85.59 (85.53,85.65) | 94.21 (94.12,94.30) |
| Physical abuse | yes | 8.23 (8.18,8.27) | 3.47 (3.40,3.55) |
|  | no | 91.85 (97.23,97.29) | 96.53 (96.45,96.60) |
| Being sent away | yes | 2.74 (2.71,2.77) | 0.38 (0.36,0.41) |
|  | no | 97.26 (97.23,97.29) | 99.23 (99.20,99.27) |
